# Prevalence and Postpartum Screening Practice for Type 2 Diabetes Following Gestational Diabetes (GDM) in a Tertiary Care Center in Western Saudi Arabia: A Three-Year Retrospective Cohort Study

**DOI:** 10.7759/cureus.75691

**Published:** 2024-12-14

**Authors:** Daniyah Alfitni, Maysaa Ageel, Ebtesam Alsulami, Abdullah Alzahrani

**Affiliations:** 1 Department of Family Medicine, King Abdulaziz Medical City, Ministry of National Guard Health Affairs, Jeddah, SAU; 2 Department of Clinical Sciences, Fakeeh College of Medical Sciences, Jeddah, SAU

**Keywords:** gestational diabetes, postpartum diabetes, risk factors, saudi, screening

## Abstract

Objectives

This study analyzed the practices and findings on postpartum type 2 diabetes mellitus (T2DM) screening among pregnant women with gestational diabetes mellitus (GDM).

Methods

A retrospective cohort study was conducted at a tertiary care center in Western Saudi Arabia, between January 1, 2016, and December 31, 2018. It involved 642 nondiabetic women with a confirmed diagnosis of GDM, who were followed until delivery. Sociodemographic and baseline clinical data, as well as data on GDM and postpartum diabetes screening, were collected from the hospital's electronic records. The incidence of T2DM following GDM was calculated as the percentage of screened participants with a positive postpartum diagnosis, along with 95% CI. Factors associated with T2DM were analyzed using Chi-square or Fisher’s exact tests, with significance set at p<0.05.

Results

The sample consisted of 642 women, primarily young and of Saudi nationality, with a notable high-risk profile including prevalent overweight and obesity (87.7%), multiparity (42.7% having four parities or more), and a frequent family history of diabetes (33.3%). Screening practices showed a great disparity between the proportion of women ordered for screening (466, 72.5%) and those effectively screened (130, 20.2%). Women who had cesarean sections were more likely to take the screening (25.0%) compared with those who had spontaneous vaginal delivery (SVD) (16.5%) (p=0.023). The incidence of post-GDM T2DM among screened participants was estimated at 13.9% (18 among 130). The incidence of post-GDM T2DM increased significantly among women with a history of three or more GDM pregnancies (50% vs. <12.5%; p=0.033) compared to their counterparts, respectively. Post-GDM T2DM was also associated with SVD (20.6% vs. 7.6%) compared to cesarean section, respectively (p=0.042). No further demographic or clinical factors were shown to be significantly associated with screening or postpartum diabetes.

Conclusions

There is a substantial gap in screening, combined with a high incidence of postpartum diabetes, among women with GDM attending our center. This highlights the urgent need for improved screening efforts, utilizing a risk-stratified approach to facilitate early detection and intervention, which could enhance long-term health outcomes.

## Introduction

The world is facing a worrying and constant rise in type 2 diabetes mellitus (T2DM) burden. As of 2021, 536.6 million individuals were affected worldwide, and this is expected to rise to 783.2 million by 2045 [1]. In Saudi Arabia, the prevalence of T2DM ranks among the highest globally, with an estimated seven million individuals diagnosed and more than three million considered pre-diabetic. This places the country as the second highest for diabetes prevalence in the Middle East and seventh worldwide [2]. Studies from the Gulf region, including Saudi Arabia, have linked T2DM with significant rates of severe complications, such as cardiovascular diseases (17.3%) and chronic kidney disease (44.3%) [3]. Furthermore, a 2022 Saudi study highlighted a progressive increase in healthcare costs associated with T2DM, especially those related to cardiovascular and renal complications [4].

The escalating T2DM burden globally is attributed to several factors, necessitating a comprehensive preventive strategy to address all modifiable risk factors and their complex interactions with the disease. Among the predisposing factors, gestational diabetes mellitus (GDM) is considered one of the strong risk factors for T2DM. According to two independent meta-analyses, published in 2020 [5] and 2021 [6], women with GDM have an 8.9- to 9.5-fold higher risk of developing T2DM. Both meta-analyses highlighted the significance of ethnicity as a cofactor for postpartum T2DM in GDM. Besides, GDM is associated with obesity, cardiovascular disease, and impaired glucose metabolism, further increasing T2DM risk [7,8]. Additionally, GDM heightens the risk of gestational hypertension, pre-eclampsia, and cesarean delivery [9], leading to dual health and economic burden [3,4,10].

The association of GDM with T2DM constitutes a serious concern for women’s health and well-being, notably given the parallel rise in GDM. In Saudi Arabia, a meta-analysis published in 2021 estimated the prevalence of GDM at 15.5%, making it one of the most highly impacted countries globally [11]. This underscores the need for specific measures to prevent and treat GDM to reduce postpartum T2DM and its societal burden. This involves timely diagnosis, rigorous GDM management, and strict postpartum glucose monitoring, especially in women with personal or familial metabolic risk factors [12,13]. This further implies determining high-risk women among GDM cases to enhance screening practices and timely management.

However, despite Saudi Arabia facing the dual burden of T2DM and GDM, data on the prevalence and screening practices for postpartum T2DM remain scarce in the country. Thus, the present study explored postpartum T2DM screening practices among women with GDM and estimated the prevalence of T2DM diagnoses among those screened. It also analyzed sociodemographic and clinical factors associated with the development of T2DM following GDM. Understanding these factors is crucial for developing targeted interventions to reduce the transition from GDM to T2DM, ultimately improving maternal and child health outcomes.

## Materials and methods

Design and participants

A retrospective cohort study was carried out at King Abdulaziz Medical City (KAMC), Ministry of National Guards Health Affairs, Jeddah, Saudi Arabia. It involved all pregnant women who attended the GDM clinic of KAMC from January 1, 2016, to December 31, 2018. Ethical approval was obtained from the institutional review board of KAMC (RJ19/026/J) on February 20, 2019.

Eligibility criteria

The study included women with a confirmed diagnosis of GDM documented in the clinic's records, who were followed until delivery. We excluded women with prior T2DM or pre-diabetes, those missed for follow-up or without medical records, deliveries outside KAMC, impaired oral glucose tolerance test (OGTT) results, and cases without GDM confirmation within the hospital's system. Women with prediabetes were excluded to avoid confounding from pre-existing impaired glucose metabolism and the potential bias introduced by their specific follow-up, monitoring, and treatment, which could influence the management of GDM and postpartum screening practices. Both GDM and T2DM were diagnosed using the classification of the American Diabetes Association [14].

Data collection and outcomes

Data were retrieved from patients' electronic records using a structured Excel sheet to ensure systematic organization and facilitate data accuracy. The collected data included 1) baseline demographic and clinical data: age, nationality, comorbidities, body mass index (BMI), family history of T2DM, and other significant chronic conditions; 2) obstetric data: parity, number of pregnancies with GDM (including the current one), results of GDM screening with OGTT levels at fasting (H0), 1-hour (H1), 2-hour (H2), and 3-hour (H3) time points, and delivery mode; 3) postpartum T2DM screening: whether the screening was ordered and carried out, and screening results, including OGTT levels at fasting (H0) and 2-hour (H2) time points.

The Excel sheet was chosen for its flexibility in handling large datasets and its compatibility with subsequent statistical analyses. To ensure data accuracy, double-entry verification and validation steps were performed. Missing data and variability in record-keeping were addressed through consensus among the investigators to ensure consistency and reliability in the dataset. A unique final dataset was compiled and used for statistical analysis.

Statistical methods

Statistical analysis was performed with the Statistical Package for Social Sciences version 21.0 for Windows (SPSS Inc., Chicago, IL, USA). Categorical variables are presented as frequency and percentage, while continuous variables are presented as mean ± standard deviation (SD). The incidence of T2DM post-GDM was calculated as the percentage (with 95% CI) of the participants who had a positive postpartum diagnosis among those who were screened. The correlation between glucose levels at different OGTT times in GDM screening (H0, H1, H2, and H3) and T2DM screening (H0 and H2) were analyzed using Pearson's correlation with the calculation of the coefficient "r." Factors associated with positive T2DM post-GDM were analyzed after excluding the participants who had no screening data; analysis used Chi-square or Fisher’s exact test, as applicable. A p-value of <0.05 was considered to reject the null hypothesis.

## Results

Baseline demographic and clinical data

A total of 903 women were diagnosed with GDM during the study period. Of these, 44 had a history of pre-diabetes or T2DM, and 217 were either lost to follow-up or had significant missing data, and were excluded. Consequently, 642 eligible women were included in the study. Of the 642 participants, 422 (65.7%) were below 35 years old and the quasi majority (634, 98.8%) were Saudi nationals. We observed low frequencies of comorbidities, involving 80 (12.4%) participants, with the most common condition being thyroid dysfunction (38, 5.9%). Majority of the participants were overweight (199, 31.0%) or obese (364, 56.7%), prior to pregnancy. Family history showed high frequencies of T2DM (214, 33.3%) and hypertension (93, 14.5%), noting that data was not available for 217 (33.8%) participants (Table 1).

**Table 1:** Baseline demographic and clinical data (N=642) ^1 ^Other comorbidities included antiphospholipid antibody syndrome (3), Hodgkin lymphoma (2), breast cancer (2), single kidney (1), dyslipidemia (1), sickle cell anemia (1), nephrectomy (1), post-thyroidectomy (1), and autoimmune hepatitis (1). BMI: body mass index; CVD: cardiovascular disease; PCOS: Polycystic ovary syndrome; T2DM: type 2 diabetes mellitus

Parameter	Level	Frequency	Percentage
Age category (years)	Below 35	422	65.7
	Above 35	220	34.3
Nationality	Saudi	634	98.8
	Non-Saudi	8	1.2
Comorbidities	Hypertension	3	0.5
	CVD	7	1.1
	PCOS	1	0.2
	Thyroid dysfunction	38	5.9
	Depression	5	0.8
	Asthma	10	1.5
	Epilepsy	5	0.8
	Other^1^	13	2.0
No. comorbidities	None	562	87.5
	1	78	12.1
	2	2	0.3
BMI	Normal	70	10.9
	Underweight	7	1.1
	Overweight	199	31.0
	Obese	364	56.7
	Not documented	2	0.3
Family history of T2DM	No	211	32.9
	Yes	214	33.3
	Not documented	217	33.8
Other family history	No	329	51.2
	Not documented	217	33.8
	Yes	96	15.0
Reported family history	Hypertension	93	14.5
	Hydrocephaly	1	0.2
	Sickle cell disease	2	0.3
	Thromboembolic disorder	1	0.2
Parameter	Unit	Mean	SD
Weight	kg	76.96	16.21
Height	cm	155.31	13.37

Obstetric history

A high proportion of the participants were multiparous, having 3 (17.4%) or 4+ (42.7%) parities, and 146 (22.8%) had at least one previous pregnancy with GDM, while 440 (68.5%) were experiencing their first GDM episode. The current GDM was managed using insulin, metformin, or lifestyle measures only in 47 (7.3%), 8 (1.2%), and 584 (91.0%), respectively; and data was missing in 3 (0.5%) participants. Delivery was marked by a high frequency of cesarean section (260, 40.5%) (Table 2).

**Table 2:** Obstetric history and post-GDM type 2 diabetes screening practice (N=642) ^§^Includes one participant who missed postpartum screening and was diagnosed later. C/S: C-section; GDM: gestational diabetes mellitus; SVD: spontaneous vaginal delivery

Parameter	Level	Frequency	Percentage
Parity	1	141	22.0
	2	115	17.9
	3	112	17.4
	4+	274	42.7
No. pregnancies with GDM	1	440	68.5
	2	107	16.7
	3+	39	6.1
	Not documented	56	8.7
Management of GDM	Insulin	47	7.3
	Metformin	8	1.2
	Lifestyle (diet or exercise)	584	91.0
	Missing data	3	0.5
Delivery mode	SVD	376	58.6
	C/S	260	40.5
	Missing data	6	0.9
Postpartum screening for T2DM	Not ordered	176	27.4
	Ordered but not done	336	52.3
	Ordered and done	130	20.2
Screening time	Less than 6 weeks	73	11.4
	More than 6 weeks	55	8.6
	Missing data	2	0.3
	Not applicable	512	79.8
T2DM screening results	No	110	17.1
	Yes^§^	18	2.8
	Unknown	514	80.1

Postpartum screening

Postpartum screening for T2DM was ordered for 466 (72.5%) of the women but was effectively carried out in only 130 (20.2%), less than six weeks after delivery in 73 (11.4%), and more than six weeks in 55 (8.6%) (Table 2). Among the analyzed factors, only delivery mode was significantly associated with effective screening (p=0.023). That is, participants who had cesarean sections were more likely to take the screening (25.0%) compared with those who had spontaneous vaginal delivery (SVD) (16.5%). SVD was associated with a higher likelihood of screening not being ordered (29.8% vs. 24.2%) or being ordered but not completed (53.7% vs. 50.8%) compared to cesarean section, respectively (results not presented in tables).

Incidence of post-GDM T2DM

Postpartum T2DM screening was positive in 18 (2.8%) participants and negative in 110 (17.1%), while data was not available for the remaining 514 (80.1%); either because the screening was not done (512, 79.8%) or screening data was not available (2, 0.3%) (Table 2). Results from OGTT in GDM and T2DM screening during the last pregnancy are presented in Table 3. These showed mean (SD) glycemia levels in GDM screening at H0 (5.11 (0.83)), H1 (11.16 (1.41)), and H 2 (10.50 (3.95)) mmol/L, noting a remarkable increase in the variance at H1 and H2. Regarding T2DM screening, mean (SD) levels at H0 and H1 were 5.35 (0.71) and 6.82 (2.13) mmol/L, respectively. Thus, by excluding cases with unknown status (N=512), the estimated incidence of T2DM following GDM among the screened participants (N=130) is 13.9% (95% CI: 8.4%-21.0%).

**Table 3:** OGTT in GDM and T2DM screening during the last pregnancy OGTT: oral glucose tolerance test; GDM: gestational diabetes mellitus; T2DM: type 2 diabetes mellitus

Screening	Time	N	Min	Max	Mean	SD
GDM	H0	546	3.60	12.40	5.11	0.83
	H1	545	6.60	18.50	11.16	1.41
	H2	544	4.70	94.00	10.50	3.95
	H3	542	3.10	17.10	7.95	1.80
T2DM	H0	130	3.40	9.50	5.35	0.71
	H2	130	3.00	16.50	6.82	2.13

Correlations between OGTT in GDM and T2DM screening

Glucose levels at H0 in GDM screening exhibited a weak positive correlation with levels at H1 (Pearson’s correlation coefficient r=0.34; p<0.01) and a very weak positive correlation with levels at H3 (r=0.10; p<0.05). A similar weak positive correlation was observed during T2DM screening between H0 and H2 (r=0.34; p<0.01). More notably, H0 levels in T2DM screening negatively correlated with GDM H1 (r=-0.25; p<0.01) and GDM H2 (r=-0.26; p<0.05), both being weak relationships. However, no significant correlation was found between T2DM H2 levels and GDM glycemia levels (Table 4).

**Table 4:** Correlations between OGTT in GDM and T2DM screening **Correlation is significant at the 0.01 level (2-tailed). *Correlation is significant at the 0.05 level (2-tailed). GDM: gestational diabetes; T2DM: type 2 diabetes mellitus; OGTT: oral glucose tolerance test

Test and time	GDM H0	GDM H1	GDM H2	GDM H3	T2DM H0	T2DM H2
GDM H0	-	-	-	-	-	-
GDM H1	0.34**	-	-	-	-	-
GDM H2	0.04	0.15**	-	-	-	-
GDM H3	0.10*	0.13**	0.25**	-	-	-
T2DM H0	0.17	-0.25*	-0.26**	-0.13	-	-
T2DM H2	0.10	0.03	0.10	0.17	0.34**	-

Factors associated with T2DM post-GDM

The incidence of T2DM was significantly higher (50%) among participants with 3+ GDM pregnancies, including the current one, compared to those with 1 (12.0%) or 2 (12.5%) GDM pregnancies (p=0.033). Furthermore, SVD mode was associated with 20.6% of cases of post-GDM T2DM compared with cesarean section (7.6%), and the difference was statistically significant at p=0.042. Age, nationality, comorbidities, thyroid dysfunction, BMI, family history of T2DM, other family history, GDM management strategy, and parity showed no significant association with the incidence of T2DM post-GDM (Table 5).

**Table 5:** Factors associated with T2DM post-GDM (N=130) *Statistically significant result (p<0.05). Test used: Fisher’s exact test; otherwise, the Chi-square test was used. BMI: body mass index; C/S: C-section; GDM: gestational diabetes mellitus; SVD: spontaneous vaginal delivery; T2DM: type 2 diabetes mellitus

Parameter	Level	No T2DM		T2DM		p-value	Statistics
		N	%	N	%		
Age category (years)	Below 35	70	87.5	10	12.5	0.574	0.32
	Above 35	42	84.0	8	16.0		
Nationality	Saudi	110	85.9	18	14.1	1.000^F^	0.33
	Non-Saudi	2	100.0	0	0.0		
Any comorbidity	No	93	86.1	15	13.9	1.000^F^	0.001
	Yes	19	86.4	3	13.6		
Thyroid dysfunction	No	103	87.3	15	12.7	0.372^F^	1.38
	Yes	9	75.0	3	25.0		
BMI	Normal or underweight	9	90.0	1	10.0	0.927	0.15
	Overweight	38	86.4	6	13.6		
	Obese	65	85.5	11	14.5		
Family history of T2DM	No	41	80.4	10	19.6	0.499	0.46
	Yes	36	85.7	6	14.3		
Other family history	No	62	84.9	11	15.1	0.323	1.09
	Yes	15	75.0	5	25.0		
Parity	1	31	96.9	1	3.1	0.207	4.56
	2	17	81.0	4	19.0		
	3	20	87.0	3	13.0		
	4+	44	81.5	10	18.5		
No. pregnancies with GDM	1	81	88.0	11	12.0	0.033*	6.85
	2	21	87.5	3	12.5		
	3+	3	50.0	3	50.0		
GDM management	Insulin	10	76.9	3	23.1	0.540	1.23
	Metformin	2	100.0	0	0.0		
	Lifestyle changes	97	86.6	15	13.4		
Delivery mode	SVD	50	79.4	13	20.6	0.042*	4.58
	C/S	61	92.4	5	7.6		

Likewise, no associations were observed between the incidence of T2DM and OGTT glycemia levels during GDM screening at H0, H1, or H2 (p>0.050) (Table 6).

**Table 6:** Association of T2DM post-GDM with OGTT glycemia levels (N=130) *Statistically significant result (p<0.05). Test used: independent t-test. GDM: gestational diabetes mellitus

Glycemia time	Level	Mean	SD	Mean	SD	p-value	t-Statistics
GDM H0	mmol/L	5.09	0.65	5.22	0.59	0.507	-0.67
GDM H1	mmol/L	11.22	1.31	11.25	1.34	0.949	-0.06
GDM H2	mmol/L	10.20	1.65	10.54	1.28	0.486	-0.70

## Discussion

Study significance and summary of findings

In a context where T2DM is escalating globally, notably in Saudi Arabia, the link between GDM and the subsequent development of T2DM is of paramount public health significance. This retrospective study analyzed the practices and findings of T2DM screening after GDM in 642 pregnant women attending a referral center in Saudi Arabia. The sample, involving predominantly young and Saudi-nationality women, showed a notable high-risk profile with prevalent overweight and obesity, multiparity, and a tangible familial burden of T2DM. The findings expose a critical gap in postpartum T2DM screening practices, with a great disparity between the proportion of women ordered for screening (72.5%) and those effectively screened (20.2%). The incidence of post-GDM T2DM among screened participants was estimated to be 13.9%, associated with multiple GDM pregnancies and SVD. No further demographic or clinical factors were shown to be significantly associated with T2DM post-GDM. This research has several key implications for clinical and public health action.

Inadequate rates of effective screening: a considerable missed opportunity

The present study highlights a critical gap in postpartum screening practices for T2DM among GDM patients, showing that only 20.2% of the concerned women are effectively screened among 72.5% who benefit from the screening order. These findings expose a missed opportunity in intercepting the progression from GDM to T2DM at a juncture where intervention could significantly alter disease trajectory and outcomes. A 2017 French study analyzed T2DM screening changes after the 2010 guidelines promoting early postpartum screening. Comparing 2007 and 2013, early screening rates (three months post-delivery) remained low at 18.5%. However, compliance at one year post-delivery increased to 67% in 2017 from 54% in 2013, indicating a need for multimodal interventions [15]. A systematic review of 27 Asian studies showed that screening uptake in Asian women varied from 13% to 82% and postpartum diabetes incidence from 3% to 58% [16]. Similar observations are found in studies from the USA and Australia, showing postpartum diabetes screening rates ranging from 19% to 73%, with some studies noting an increase in compliance over time [17].

Enhancing postpartum T2DM screening involves addressing potential issues and barriers in all screening phases. The initial phase includes ordering and scheduling the test by the provider. A survey by Keely et al. showed both patients and primary care physicians believe the prescriber and provider are responsible for postpartum screening [18]. A 2014 systematic review found variability in practices influenced by physician specialty and care center type, with high-risk pregnancy settings showing better practices. Nonetheless, the review noted that good practice was not predictive of effective screening uptake [17]. This is consistent with our findings, which reveal that effective screening was conducted for only 20% of the patients, despite orders being placed for 72.5%. This underscores the influence of other factors in the post-ordering phase.

The next phase includes factors affecting women's ability or motivation to attend screening tests. A systematic review of 16 qualitative studies identified two main categories of factors influencing the attendance of screening: healthcare system-related and patient-related factors. Healthcare system-related factors are categorized into two major dimensions. The first concerns the quality of provider-patient communication and relationship conditioning women’s ability to understand both the necessity and procedure of the screening. The second aspect includes logistic factors, such as screening scheduling and process, which influence the practicality of the test uptake. Patient-related factors include cognitive and behavioral factors, such as awareness and concerns about health and specific health risks and care-seeking behavior, in addition to family-related factors such as maternity issues, support, and professional and other responsibilities [19]. Many patients struggle with attending screenings and follow-ups due to motherhood issues and inadequate awareness about GDM and postpartum diabetes risks [20]. These factors highlight the need for comprehensive strategies focusing on the overall patient experience.

Recommendations to improve screening uptake

Given the complexity of the issue, improving screening uptake requires multifaceted strategies to address practical, educational, and systemic barriers. Interventions targeting patient-related factors, including educational programs, counseling, and reminders about postpartum screening have been effective in enhancing screening uptake [17]. However, the somewhat limited effectiveness of these strategies, when implemented separately, underscores the need for a multimodal approach. This involves combining several interventions for the same patient, aiming to maximize the overall impact on screening uptake [21]. By integrating various strategies, healthcare providers can address a broader range of barriers, involving the previously discussed phases of the screening process, while tailoring action to the patient’s unique needs and preferences. Moreover, enhancing healthcare provider engagement and system-wide changes are critical. The International Federation of Gynecology and Obstetrics (FIGO) emphasizes clinician education, recall systems, standardized protocols, and patient-centered practices to enhance screening. Making clinics more welcoming, personalizing test accessibility, and integrating glucose testing with routine check-ups improve attendance and patient experience. Educating women on screening importance and addressing misconceptions are vital [19]. These measures, in the Saudi context, imply that evaluating physicians' engagement, preparedness, and self-efficacy, as well as identifying systemic obstacles, can help inform targeted strategies to improve screening practices. Such efforts would complement broader system-wide changes and enhance the overall effectiveness of postpartum screening initiatives. Applying these measures in the Saudi context implies assessing barriers to screening among healthcare providers, including their engagement, preparedness, and self-efficacy, along with organizational and healthcare system-related factors. Understanding these factors is crucial to developing targeted strategies that improve screening practices.

Furthermore, the consistent challenge in implementing early screening, four to 12 weeks after discharge, indicates the need for more innovative methods and convenient screening strategies. Some studies explored immediate postpartum screening (one to five days), showing 81% sensitivity, 61% specificity, and a negative likelihood ratio of 0.3 [22]. Despite limited performance, this approach may complement traditional methods, particularly for high-risk nonadherent patients, and presents opportunities to develop more sensitive screening techniques immediately after childbirth, thus maximizing uptake.

Incidence and risk factors of T2DM post-GDM

The incidence of post-GDM T2DM among screened participants was estimated at 13.9%, corresponding to 18 individuals in the cohort. However, when considering the non-screened individuals, the theoretical total of T2DM cases rises to 64, indicating that 46 cases were potentially missed for diagnosis. In other terms, for every woman diagnosed with postpartum diabetes, approximately three remain undiagnosed. This exposes to delayed diagnosis, inadequate management, and diabetes complications. Such a gap highlights the urgent need for enhanced screening efforts to ensure early detection and intervention, ultimately improving long-term health outcomes for these women.

A systematic review focusing on Asian women found that the transition to T2DM among women with GDM ranged widely from 3% to 58% [16]. However, data from the Middle East showed incidence figures relatively comparable to our findings, including 20.6% in the United Arab Emirates [23] and 18.9% in Turkey [24]. This great variability in T2DM rates across the studies can be attributed to differences in diagnostic criteria for GDM and T2DM, the type of study, population demographics, and follow-up duration. The postpartum glucose testing protocols also varied, with some using OGTT exclusively and others including random plasma glucose or fasting blood sugar, impacting detection. Furthermore, the use of routine care or complementary approaches, such as SMS reminders, and adherence to follow-up protocols differed across studies. Finally, sociocultural and ethnic factors might also contribute to the variability. Collectively, these methodological and contextual differences explain the wide range of diabetes rates observed in the studies.

In addressing the challenge of postpartum diabetes and screening uptake, developing a risk stratification strategy seems a plausible approach. This approach requires identifying key risk factors for GDM to T2DM transition and determining profiles linked to individual or socioeconomic vulnerabilities that contribute to inadequate screening uptake. The present study identified two risk factors for T2DM post-GDM including recurrent GDM and SVD. Recurrent GDM, a distinct clinical entity affecting approximately 50% of women with GDM, is associated with an increased risk of postpartum T2DM [25,26]. Therefore, women with recurrent GDM should receive special attention for screening and management. Regarding the mode of delivery, we observed a lower incidence of T2DM among women who underwent cesarean sections, combined with a significantly greater likelihood of screening. This may stem from discrepancies in care and adherence levels to care plans, contributing to a higher risk of developing postpartum diabetes in women who have SVD.

The literature shows various risk factors for GDM progression to T2DM, suggesting a multifactorial pathophysiology. Anthropometric factors including waist and hip circumferences, BMI, excessive weight gain during pregnancy, and difficulty losing weight post-delivery were all associated with a high risk for T2DM development [27-29]. Our cohort was characterized by a high frequency of obesity and a family history of T2DM, both known to elevate the risk of postpartum T2DM [7,8,30]. This highlights the necessity for stricter T2DM screening measures among Saudi women with GDM due to their high-risk metabolic profile.

Women who necessitate oral hypoglycemic drugs or insulin to manage their GDM are also at higher risk for postpartum diabetes [27]. Further data showed that fasting and OGTT glycemia levels, gestational age at GDM diagnosis, and shorter breastfeeding duration were significantly associated with postpartum diabetes [27-29]. Conversely, the present study showed no statistically significant association of T2DM risk with the treatment regimen or OGTT levels. Identifying these risk factors enables adapting GDM management and diabetes screening strategies, stratified by risk level.

Limitations

The present study is principally limited by information bias and lack of key data, inherent to its retrospective design. These limitations impeded the availability and analysis of the significant sociodemographic and clinical factors associated with screening uptake and postpartum diabetes. The low proportion of screened women has further weakened the power of subsequent comparative analysis. Furthermore, the number of patients with missing data or lost for follow-up (n=217) represents 25% of eligible women and may induce a selection bias where women with poor outcomes are more likely to adhere to follow-up, and conversely, potentially affecting the estimation of screening rates and post-GDM T2DM incidence.

## Conclusions

This study highlights a significant gap in postpartum T2DM screening, showing that while three-quarters of women were ordered for screening, only one-fifth were effectively screened. The incidence of type 2 diabetes post-GDM among those screened was found to be 13.9%, aligning with figures from Middle Eastern countries. When accounting for non-screened individuals, we estimate that for every woman diagnosed, approximately three remain undiagnosed. This worrying figure highlights the urgent need for improved screening efforts to facilitate early detection and intervention, which could enhance long-term health outcomes.

The study identified recurrent GDM and SVD as risk factors for postpartum diabetes, highlighting the need for a thorough exploration of socioeconomic and clinical risk factors for both screening and postpartum diabetes. This also highlights the potential benefits of a risk-stratified approach to screening and management.
